# Virtual Lifelong Learning Among Older Adults: Usage and Impact During the COVID-19 Pandemic

**DOI:** 10.7759/cureus.24525

**Published:** 2022-04-27

**Authors:** Brittany Derynda, Joy Siegel, Linda Maurice, Nicole Cook

**Affiliations:** 1 Public Health, Nova Southeastern University Dr. Kiran C. Patel College of Osteopathic Medicine, Fort Lauderdale, USA; 2 Public Health, Sursus Impact Group, Lifelong Learning Institute, Fort Lauderdale, USA

**Keywords:** technology- enhanced connections, older adults, social isolation, covid-19 pandemic, senior companionship

## Abstract

Social isolation and loneliness are major health concerns for older adults, with the current prevalence of social isolation among older adults estimated to be as high as 43%. In older adults, loneliness and social isolation have both been linked with poor health outcomes including falls, re-hospitalizations, dementia, and all-cause mortality. During the coronavirus disease 2019 (COVID-19) pandemic, older adults constituted one of the most at-risk groups and were faced with some of the strictest and earliest social distancing recommendations, which were associated with increased feelings of loneliness and increased rates of depression and anxiety, upwards of 12%. The objective of this study was to identify the impact of online social connection on feelings of isolation and companionship among older adults during the COVID-19 pandemic. Following the Centers for Disease Control and Prevention (CDC) guidelines in March 2020, two South Florida social and educational programs for older adults adopted online programming utilizing the Zoom platform. A research team worked collaboratively with senior stakeholders to develop and administer a survey to understand the impact of online social connections on feelings of social isolation. One year later in 2021, the survey was reviewed, modified, and re-administered. Respondents of the survey included 211 older adults (mean age 75.5 years old). Notable findings included a strong association between frequency of online class attendance and increased feelings of connectedness (p<0.001), improved spirits (p<0.001), and decreased feelings of social isolation (p<0.001). These results underscore the importance and contribution of online programming among older adults during times of social isolation. Overall, clinical practitioners should consider the importance of initiating discussions with older adults regarding returning to activities that they enjoyed prior to the COVID-19 pandemic.

## Introduction

Social isolation and loneliness are major health concerns for older adults, even prior to the coronavirus disease 2019 (COVID-19) pandemic [1]. It is estimated that the current prevalence of social isolation among community-dwelling older adults is as high as 43% [2]. In older adults, loneliness and social isolation have both been linked with poor health outcomes, including falls, re-hospitalizations, dementia, and all-cause mortality [3]. Berkman established a framework showcasing the three ways in which individuals may be negatively impacted by limited social networks including health behavior, psychological, and physiological [4].

During the COVID-19 pandemic, older adults constituted one of the most at-risk groups and were faced with some of the strictest and earliest physical distancing recommendations. While recommendations to reduce face-to-face contact were vital to decrease rates of COVID-19 infection transmission, social distancing (especially among older adults) was demonstrated to be associated with increased feelings of loneliness and increased rates of depression and anxiety, upwards of 12% [5]. In response to these difficulties experienced by older adults, many organizations and support groups seized the opportunity to develop and adopt technology to improve connectivity and keep individuals engaged, especially after the Centers for Disease Control and Prevention (CDC) established social distancing guidelines and recommendations during March 2020. In an effort to better understand the effectiveness of programs to address social isolation among older adults during the COVID-19 pandemic, we aimed to identify the impact of online social connection on feelings of isolation and companionship among seniors during the COVID-19 pandemic.

## Materials and methods

Survey development and promotion

The study population included older adults from two social and educational programs in South Florida described below. Both programs have a shared goal of providing weekly education, and social and fitness opportunities to support social networks and healthy aging among seniors. One program, the Lifelong Learning Institute (LLI) affiliated with Nova Southeastern University (NSU), services older adults, mostly retired professionals (with an average of 75 years) within the South Florida region and has for over 25 years. The other program is a South Florida regional health program that provides insurance to over 20,000 regional members over the age of 65. The health program provides access to various wellness programs that promote nutrition, exercise, and healthy life habits. Following the social distancing guidelines from the CDC established in March 2020 these two programs were rapid adopters of the Zoom platform, a software program used for teleconferences, for program delivery. Monthly schedules were established by the program coordinators in March 2020 and members received training on utilization of virtual technology to improve engagement.

In May 2020, the study team designed a survey that was to be administered to the LLI group’s members to investigate the impact that online social connections had on feelings of social isolation and adherence to social distancing regulations. Questions from the survey were focused on feelings of isolation and companionship, adherence to social distancing regulations, and comfortability using technology for online programming. All the questions in the final survey were adopted from the Technology Acceptance Model [6], Wave 3 National Social Life, Health and Aging Project (NSHAP) questionnaire [7], and Healthy Days Core Module [8]. The research team (composed of researchers, medical and undergraduate students, and nine advisory members) met via the Zoom platform three times and modified questions to develop a final 31-question survey. The survey was distributed to the 170 members who were enrolled in LLI programming via an email containing the survey link sent directly from the group’s advisory team. There was a follow-up email sent one week later to remind members to complete the survey. Study data were collected and managed using the research electronic data capture (REDCap) program hosted at NSU [9,10]. REDCap is a secure, web-based software platform designed to support data capture for research studies, providing 1) an intuitive interface for validated data capture, 2) audit trails for tracking data manipulation and export procedures, 3) automated export procedures for seamless data downloads to common statistical packages, and 4) procedures for data integration and interoperability with external sources.

In May 2021, the study team (which now included a programming director from the South Florida regional health plan) met to review and revise the original survey to understand the impact of the pandemic on social isolation one year later. The revised study contained eight of the original questions from the survey in May 2020 that had significant findings. The focus of this survey was on the impact of online social connection on the mental health of older adults engaging in Zoom platform programming, and because of this, 16 questions were omitted from the original survey that focused on comfortability using technology. In addition to the focus of the survey, these questions were omitted to keep the length of the survey short in hopes to increase participation. New questions were added to assess the extent to which respondents were social distancing and their vaccination status to investigate a possible relationship between these factors and engagement in online social connections. The final survey included 21 questions. The research protocol was approved by the NSU’s institutional review board (IRB) and a waiver of consent was obtained from the IRB (approval number: IRB No. 2020-244-Web). In May 2021, the survey was distributed to a combined 1,035 members from both the LLI and the regional health plan via an email containing the survey link sent from the leadership of each organization. There was a follow-up email sent one week later to remind members to complete the survey. Study data were collected and managed using the REDCap program hosted at NSU [9,10].

Data analysis

Descriptive statistics and chi-square analysis were completed using Microsoft Excel version 16.54 for all respondents. A subgroup analysis was conducted among responders to assess trends in select questions from May 2020 to the May 2021 survey.

## Results

May 2020 survey results

Respondents of the first survey included 127 members from the LLI, mean age of 75.5 years, with a 74.7% response rate. In addition, 62.5% of respondents identified as female. Among participants, those who reported that they were not tech-savvy compared to those who reported that they were tech-savvy had 8.3 times higher odds of feeling less companionship (p<0.05). Furthermore, those who isolated alone compared to those who isolated with someone else had 6.7 times higher odds of feeling less companionship (p<0.05).

May 2021 survey demographics

Respondents of the second survey included 211 members from both the LLI and the South Florida regional health plan, mean age of 75.5 years with a range of 32 to 98 years (Table 1).

**Table 1:** Demographics of the participants. COVID-19: coronavirus disease 2019

	N	Percent
Male	58	27.5
Age		
45-64	21	9.9
65-74	79	37.3
75-84	89	41.9
85+	23	10.8
Fully vaccinated against COVID-19	199	94.3

Among the 211 members, 20.39% responded to the survey. In addition, when further stratified by group, the response rate of the LLI was 52.35% and the response rate of the South Florida regional health plan was 10.75%. The sample was composed of an even distribution from both groups being 42.2% and 44.1% with 6.6% stating they belonged to both groups or selected no affiliation and were not included in the analysis. Most of the respondents were female (72.6%). In terms of vaccination status, 91.9% reported completing two doses of one of the two-dose COVID-19 vaccinations available, 2.8% had completed one dose of a two-dose vaccine and 2.4% had completed one dose of the one-dose vaccine.

Frequency of online engagement

There was a significant association between the frequency of online social connection (once or several times a week compared to less than once a week or once a month) and reporting that the Zoom platform was a good tool to keep spirits up (odds ratio (OR) = 17.8, p <0.001), reducing feelings of social isolation (OR= 8.7, p = 0.001), and helping to increase feelings of connectedness to others (OR= 12.1, p = 0.001) (Table 2).

**Table 2:** Chi-square results comparing frequency of online social programming participation and feelings of social isolation.

	Agree		Disagree		X^2^	P value
Attendance	N	%	N	%		
Q1: Zoom has helped me keep my spirits high during safer-at-home						
More than once per week	136	90.0%	7	50.0%	17.8	<0.001
Less than once per week	15	10.0%	7	40.0%		
Q2: Zoom has helped me keep away from feeling socially isolated during safer-at-home						
More than once per week	144	90.1%	9	64.3%	8.7	0.003
Less than once per week	15	9.4%	5	35.7%		
Q3: Zoom has helped me feel connected during safer-at-home						
More than once per week	154	89.5%	8	57.1%	12.1	0.001
Less than once per week	18	10.5%	6	42.9%		

Responses regarding vaccination status

In terms of vaccination status at the time the results were collected, 194 of the 211 respondents (91.9%) completed both doses of the two-dose vaccines, with six respondents (2.8%) completing one of the two-dose vaccines, five respondents (2.4%) completing the one-dose vaccine, and six respondents (2.8%) having not completed any doses of the vaccines.

Comparison between 2020 and 2021 responses

Among participants who completed the survey in 2020, 82.1% of respondents agreed that the Zoom platform helped keep their spirits up. This is compared to 80.6% of respondents in 2021 who agreed that the Zoom platform helped keep their spirits up. In addition, results from both 2020 and 2021 respondents showed that 83.6% of participants agreed or strongly agreed that Zoom had a positive impact on their quality of life.

## Discussion

In our study, both the 2020 and 2021 surveys revealed that increased frequency of online class attendance had a positive impact on feelings of connection, increased spirits, and decreased feelings of social isolation, demonstrating a sustained positive impact of online social connection. See the Appendices for a copy of both surveys (Table 3). Chen et al. had similar findings, demonstrating that online social connection was positively associated with well-being and quality of life. [11] These findings suggest that social programs serving seniors should continue to offer online engagement options, regardless of social distancing recommendations. Virtual programming may continue to be beneficial for individuals who are homebound, live in rural areas, who are in assisted living programs, and who are not able to attend social engagement opportunities in person. To enhance engagement in these programs, the ideal class format and comfortability using technology can be further evaluated in future research efforts.

In addition, our study demonstrated that older adults who were not comfortable using technology had 8.3 times higher odds of feeling less companionship. These findings are similar to those found by Nguyen et al., in which internet skills moderated the relationship between online social engagement and social capital [12]. As such, future research endeavors should aim to gain insight into technology use skills among older adults and effective programming.

Our study demonstrated that a majority of participants were vaccinated, as 91.9% of survey participants had completed the vaccination requirements at the time of the survey. Interestingly, however, we found that vaccination status did not impact adherence to social distancing regulations as 54.5% reported that they were staying at home nearly all the time or were only leaving home to buy essentials. This highlights the importance of initiating conversations with older adults regarding their new fears when returning to their “pre-COVID-19 pandemic” ways of living. Alternatively, it highlights the impact that the COVID-19 pandemic had on the creation of new routines in older adults that they now enjoy more than prior to the pandemic onset.

One limitation of this study is the low respondent percentage. The survey was sent via email to 1,035 individuals, 170 were members of the LLI, and 865 were members of the South Florida regional health plan. The total respondent number was 211 with a total response rate of 20.39%. Upon further stratifying the data, the response rate of LLI was found to be much higher than that of the South Florida regional health plan. Reasons for this could be attributed to LLI having a much smaller number of participants overall, in which all members are encouraged to attend the same classes. In contrast to this, the South Florida regional health plan has a very large number of participants, in which there are a variety of classes members can attend. By being in a smaller group participants may feel that they are held more accountable to complete a survey that is sent to them by a leader that they know personally and peers on the committee creating the methodology. In addition, the research group had completed a survey with the LLI the year prior and encouraged continued participation in the survey from the members, possibly leading to an increased response rate.

## Conclusions

Social isolation and loneliness are major health concerns for older adults and are linked to poor health outcomes. These concerns have increased during the COVID-19 pandemic due to strict physical distancing recommendations, especially among older adults. Two South Florida programs were forced to develop and adopt online platforms to offer continued opportunities for social engagement of older adults. The evidenced-based value of Zoom platform use among older adults and the opportunities for connection during times of social isolation cannot be denied, emphasizing the importance of adaptation of similar platforms by like-minded programs for older adults. In addition, the initiation of discussions with older adults regarding returning to activities that they enjoyed prior to the COVID-19 pandemic should be further investigated to identify the reason for the change. This study emphasizes the importance and need for the adoption of new platforms to allow continued social engagement among older adults.

## Appendices

**Table 3:** Survey questions.

Demographics	
Which group are you a member of?	LLI
	AvMed
	Both
	Neither
What is your gender?	Female
	Male
	Do not identify as female or male
What is your age?	Free response
During the majority of “stay at home” orders, who did you reside with? (Choose more than one option)	Alone
	A spouse
	A pet
	A friend
	A caregiver
	Other
This next set of questions asks about your experience with technology in terms of improving social connection and reducing social isolation.	
In the past 12 months, how often did you attend virtual meetings of any organized groups? (Examples include classes/lectures, social gatherings, a choir, a committee or board, a support group, a sport or exercise group, a hobby group, or a professional society.)	Several times a week
	Once a week
	Less than once a week
	About once a month
	Several times a year
	Less than once a year
Video technology (Zoom, Facebook, FaceTime, etc.) has helped me feel connected to others during “safer-at-home”	Strongly agree
	Agree
	Neither agree nor disagree
	Disagree
	Strongly disagree
Video technology has helped me from feeling isolated during “safer-at-home”	Strongly agree
	Agree
	Neither agree nor disagree
	Disagree
	Strongly disagree
Video technology has helped keep my spirits up during “safer-at-home”	Strongly agree
	Agree
	Neither agree nor disagree
	Disagree
	Strongly disagree
Video technology has helped improve the quality of my life during “safer-at-home”	Strongly agree
	Agree
	Neither agree nor disagree
	Disagree
	Strongly disagree
I am comfortable using Zoom to connect with family/friends	Strongly agree
	Agree
	Neither agree nor disagree
	Disagree
	Strongly disagree
In an effort to reduce the spread of COVID-19, many are practicing social distancing and self-isolation. Self-isolation is the act of staying away from situations where you may be in close contact with others, such as social gatherings, work, school, faith-based gatherings, sports gatherings, restaurants, and other public gatherings. This next set of questions asks about your experiences with COVID-19, social distancing, and self-isolation. Please keep in mind that this survey is anonymous.	
Have you personally been diagnosed with COVID-19?	No
	Yes, I was diagnosed, but effectively managed symptoms at home
	Yes, I was diagnosed, with severe symptoms and required brief hospitalization
	Yes, I was diagnosed, with severe symptoms and required ventilation
Have you tested positive for COVID-19?	Yes, I have tested positive one time (list date below)
	Yes, I have tested positive multiple times (List dates below)
	I have been clinically diagnosed as positive but have not had a positive test
	No, I have not tested positive
Have you received the vaccine for COVID-19?	I have received both doses and it has been more than 2 weeks since the second dose
	I have received both doses but it has not been more than 2 weeks since the second dose
	I received the first dose but am waiting to receive the second dose
	I received the one dose vaccination (Johnson & Johnson)
	I have not received the first vaccine
Do you have any ongoing medical condition that might put you at a higher risk for severe illness from COVID-19? Some conditions can put you at higher risk such as: cardiovascular conditions, chronic lung disease, obesity or diabetes, a weakened immune system due to smoking, cancer treatment, other immune deficiencies	Yes (Please specify below)
	No
	Don’t know
If yes, please list the medical condition that you have that might put you at a higher risk for severe illness from COVID-19	Free Response
On a scale of 1-5, how serious do you think COVID-19 is as a risk to you personally or if you contract it, to you transmitting it to your loved ones	1—Not serious
	2
	3
	4
	5—Very serious
To what extent are you self-isolating?	I am staying at home nearly all the time.
	I only leave my home to buy food and other essentials
	I leave my home to buy food and other essentials, as well as to go to restaurants and some social gatherings
	I am doing everything I normally do
To what extent does your peer group follow self-isolation guidelines?	Majority of my friends follow the guidelines
	Some of my friends follow the guidelines
	Majority of my friends do not follow the guidelines
If you are no longer self-isolating and/or social distancing, when did you stop?	Free response
If you are no longer self-isolating and/or social distancing, why did you stop? (Choose more than one option)	Wanted to see friends and family
	Peer pressure
	Work/volunteer activities
	Concern for mental health/feeling lonely
	I do not think self-isolation is effective in preventing the spread of COVID-19
	I got COVID-19 and then recovered
	I have been fully vaccinated
	Other
Did you maintain social distancing and/or self-isolation through the holiday (Christmas/Hanukkah/New Year)?	Yes
	No

## References

[1] Nicholson NR (2012). A review of social isolation: an important but underassessed condition in older adults. J Prim Prev.

[2] Ejiri M, Kawai H, Fujiwara Y, Ihara K, Hirano H, Kojima M, Obuchi S (2018). Predictors of social isolation among older people living in urban area: a prospective study. (Article in Japanese). J Stage.

[3] Coyle CE, Dugan E (2012). Social isolation, loneliness and health among older adults. J Aging Health.

[4] Berkman LF (2000). Which influences cognitive function: living alone or being alone?. Lancet.

[5] Robb CE, de Jager CA, Ahmadi-Abhari S (2020). Associations of social isolation with anxiety and depression during the early COVID-19 pandemic: a survey of older adults in London, UK. Front Psychiatry.

[6] Davis FD (1989). Perceived usefulness, perceived ease of use, and user acceptance of information technology. MIS Quart.

[7] Waite L, Cagney K, Dale W (2017). National Social Life, Health and Aging Project (NSHAP): Wave 3. ICPSR36873-v1. Ann Arbor, MI: Inter-university Consortium for Political and Social Research distributor.

[8] Moriarty DG, Zack MM, Kobau R (2003). The centers for disease control and prevention's healthy days measures - population tracking of perceived physical and mental health over time. Health Qual Life Outcomes.

[9] Harris PA, Taylor R, Thielke R, Payne J, Gonzalez N, Conde JG (2009). Research electronic data capture (REDCap)—a metadata-driven methodology and workflow process for providing translational research informatics support. J Biomed Inform.

[10] Harris PA, Taylor R, Minor BL (2019). The REDCap consortium: building an international community of software platform partners. J Biomed Inform.

[11] Chen E, Wood D, Ysseldyk R (2022). Online social networking and mental health among older adults: a scoping review. Can J Aging.

[12] Nguyen M, Hunsaker A, Hargittai E (2020). Older adults' online social engagement and social capital: the moderating role of internet skills, information, communication & society.

